# Electrical Characterization of Germanium Nanowires Using a Symmetric Hall Bar Configuration: Size and Shape Dependence

**DOI:** 10.3390/nano11112917

**Published:** 2021-10-30

**Authors:** Ahmad Echresh, Himani Arora, Florian Fuchs, Zichao Li, René Hübner, Slawomir Prucnal, Jörg Schuster, Peter Zahn, Manfred Helm, Shengqiang Zhou, Artur Erbe, Lars Rebohle, Yordan M. Georgiev

**Affiliations:** 1 Institute of Ion Beam Physics and Materials Research, Helmholtz-Zentrum Dresden-Rossendorf (HZDR), 01328 Dresden, Germany; h.arora@hzdr.de (H.A.); zichao.li@hzdr.de (Z.L.); r.huebner@hzdr.de (R.H.); s.prucnal@hzdr.de (S.P.); p.zahn@hzdr.de (P.Z.); m.helm@hzdr.de (M.H.); s.zhou@hzdr.de (S.Z.); a.erbe@hzdr.de (A.E.); l.rebohle@hzdr.de (L.R.); y.georgiev@hzdr.de (Y.M.G.); 2 International Helmholtz Research School for Nanoelectronic Network, Helmholtz-Zentrum Dresden-Rossendorf (HZDR), 01328 Dresden, Germany; 3 Institute of Applied Physics, Technical University of Dresden, 01062 Dresden, Germany; 4 Fraunhofer Institute for Electronic Nano Systems (ENAS), 09126 Chemnitz, Germany; florian.fuchs@enas.fraunhofer.de (F.F.); joerg.schuster@enas.fraunhofer.de (J.S.); 5 Center for Materials, Architectures and Integration of Nanomembranes (MAIN), Chemnitz University of Technology, 09126 Chemnitz, Germany

**Keywords:** germanium nanowires, Hall bar configuration, Hall effect, electrical characterization

## Abstract

The fabrication of individual nanowire-based devices and their comprehensive electrical characterization remains a major challenge. Here, we present a symmetric Hall bar configuration for highly p-type germanium nanowires (GeNWs), fabricated by a top-down approach using electron beam lithography and inductively coupled plasma reactive ion etching. The configuration allows two equivalent measurement sets to check the homogeneity of GeNWs in terms of resistivity and the Hall coefficient. The highest Hall mobility and carrier concentration of GeNWs at 5 K were in the order of 100 ${\mathrm{cm}}^{2}/\left(\mathrm{Vs}\right)$ and $4\times 10^{19}\;{\mathrm{cm}}^{-3}$, respectively. With a decreasing nanowire width, the resistivity increases and the carrier concentration decreases, which is attributed to carrier scattering in the region near the surface. By comparing the measured data with simulations, one can conclude the existence of a depletion region, which decreases the effective cross-section of GeNWs. Moreover, the resistivity of thin GeNWs is strongly influenced by the cross-sectional shape.

## 1. Introduction

Germanium (Ge) is a material that is most compatible with silicon (Si)-based complementary metal-oxide-semiconductor (CMOS) processes. It has a higher number of electrons and hole mobility compared to Si, leading to an improved device performance [1,2,3]. Moreover, nanowires (NWs) have been considered as an attractive building block for electronic and optoelectronic applications because of their unique properties such as a low dimensionality, quantum confinement, surface sensitivity, and low leakage current. Hence, GeNWs are promising high-mobility nanostructures for future nanoelectronics [4,5,6,7]. Despite constant improvement in the performance of semiconducting NW-based devices, evaluating electrical properties of single NWs still remains a challenging task because of their small size. To date, several techniques have been developed to this end. The field effect (FE) mobility measurement is the most commonly used technique, although it has some shortcomings [8,9,10,11,12,13,14]. The accuracy of this method depends largely on the precision of the estimated gate capacitance. Furthermore, the FE measurement characterizes only the depleted layer of charge carriers close to the gate and estimates the carrier concentration of NWs by assuming a radially constant mobility. Moreover, the FE measurement is carried out without taking into account the contact resistance, leading to an overestimation of the carrier concentration [13,14,15]. Recently, the Hall effect measurement, which is a standard method to determine transport characteristics such as carrier mobility and carrier concentration in planar semiconductors, has been applied for semiconducting NWs via four-probe and three-probe device geometries [16,17,18,19,20,21,22]. Dissimilar to the FE measurement, the Hall effect measurement provides a more precise characterization of the carrier concentration by considering the entire cross-section of the NW [15]. However, the fabrication of NW-based Hall devices with a sub-30 nm diameter is a challenging process and requires a precise alignment of the metal contacts. The Hall bar configuration with narrow bars allows the precise positioning of metal contacts on the NW sidewalls and avoids the overlapping of metal contacts, especially for thin NWs. Furthermore, the Hall bar configuration increases the accuracy of the Hall effect measurement by avoiding shorting out the Hall voltage. Recently, the Hall bar configuration has been used for silicon nanowires with a five-contact geometry [23] and for two-dimensional (2D) materials with an eight-contact geometry [24]. To the best of our knowledge, a Hall bar configuration has not been developed for GeNWs so far.

In this work, GeNWs are fabricated by a top-down approach using electron beam lithography (EBL) and inductively coupled plasma-reactive ion etching (ICP-RIE). To investigate the electrical properties of the fabricated GeNWs, we develop a symmetric six-contact Hall bar configuration. Such a configuration enables the Hall effect and four-probe resistivity measurements on the same GeNW. The narrow bars act as point-like contacts and avoid shorting out the Hall voltage caused by source current contacts, leading to high accuracy. There are some intrinsic physical mechanisms that can change the current density in NWs. Some of these effects, such as thermoelectric voltages, can be minimized by controlling the temperature of the NW vicinity. Moreover, the misalignment voltage can be eliminated by reversing the source current or the applied magnetic field during the Hall effect measurement [25]. Hence, to minimize the errors induced by intrinsic physical mechanisms, the source current is swept through the GeNWs for each applied magnetic field at low temperatures. Also, the effect of NW width on transport parameters, such as resistivity, carrier concentration, and mobility, is investigated. Furthermore, the size- and shape-dependent resistivity of the GeNWs was simulated with two different models. The measured resistivities of GeNWs are compared to simulations, indicating the existence of a scattering region near the surface.

## 2. Materials and Methods

### 2.1. Experimental Setup

**Figure 1:**
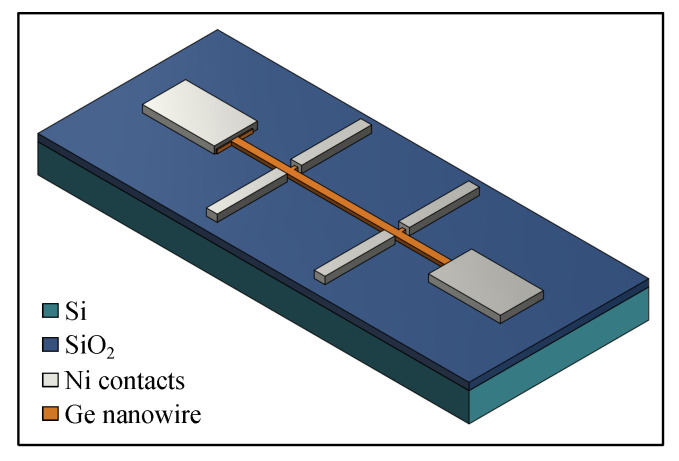
Schematic diagram of the fabricated device with the six-contact Hall bar configuration.

Highly p-type doped germanium-on-insulator (GeOI) substrates with a 38 nm thick Ge layer, $1.2\times 10^{-3}\;\Omega \cdot$cm resistivity, and 200 nm buried ${\mathrm{SiO}}_{2}$ layer were structured using EBL and ICP-RIE to fabricate GeNWs as follows. The negative tone resist hydrogen silsesquioxane (HSQ) (*Dow Corning X-1541* with original 6% concentration), which has sub-5 nm resolution, small edge roughness, and high etch resistance [26,27,28], was diluted to 2% concentration in methyl isobutyl ketone (MIBK) and spin-coated on the pre-cleaned and passivated [29] GeOI substrates at 2000 rpm for 30 s to obtain a 40 nm thick HSQ layer. Then, the samples were baked at 120 °C for 2 min and loaded into the EBL system. The electron beam exposure was carried out using a *Raith e-Line Plus* system at an accelerating voltage of 10 kV, 1000 μC/cm${}^{2}$ area dose, 30 μm aperture size, and 2 nm area step size. The samples were developed using a high-contrast tetramethylammonium hydroxide (TMAH)-based development process [30] and dried with an $N_{2}$ gun. A *SENTECH* ICP-Reactive Ion Etcher SI 500 with a continuous flow of $SF_{6}$ (10 sccm), $C_{4}F_{8}$ (22 sccm), and $O_{2}$ (5 sccm) gases at 0.9 Pa chamber pressure, 400 W ICP power, and 12 W RF power was used to transfer the HSQ patterns of NWs into the top Ge layer. At the end, the HSQ was removed by a dip in HF (1% in H_2_O) solution for 50 s. In order to fabricate the Hall contacts, EBL, metal deposition, and a lift-off process were employed. A positive tone resist, polymethyl methacrylate (PMMA), was spin-coated on the samples with the patterned GeNWs at 3000 rpm for 50 s and baked at 180 °C for 10 min. Then, the electron beam exposure was performed using the following parameters: 10 kV accelerating voltage, 120 μC/cm${}^{2}$ area dose, 20 μm aperture size, and 20 nm area step size. The exposed samples were developed in isopropanol/deionized water (7:3) solution. Right before nickel (Ni) deposition for the metal contacts, samples were placed into an acetic acid/DI water (1:7) solution to remove the native oxide [29]. An ultrahigh vacuum electron beam *BESTEC* evaporation tool was used to deposit a 50 nm thick layer of Ni. Afterwards, the lift-off was carried out in acetone. A schematic diagram of the fabricated device with the six-contact Hall bar configuration is shown in Figure 1.

Bright-field (BF) and high-resolution transmission electron microscopy (HR-TEM) imaging was performed with an image $C_{s}$-corrected Titan 80–300 microscope (FEI, Eindhoven, Netherlands) operated at an accelerating voltage of 300 kV. Prior to TEM analysis, the specimen mounted in a double-tilt low-background holder was placed for 8 s into a Model 1020 Plasma Cleaner (*Fischione*, Export, PA, USA) to remove possible contamination. The preparation of the TEM specimen containing the cross-section of the GeNW was conducted by in situ lift-out using a Helios 5 CX focused ion beam (FIB) system (*Thermo Fisher*, Waltham, MA, USA). To protect the GeNW surface, a carbon cap layer was deposited at the beginning with electron-beam-assisted and, subsequently, followed by Ga-FIB-assisted precursor decomposition. Afterward, the TEM lamella was prepared using a 30 keV Ga FIB with adapted currents. Its transfer to a 3-post copper lift-out grid (*Omniprobe*) was performed with an EasyLift EX nanomanipulator (*Thermo Fisher*). To minimize sidewall damage, Ga ions with 5 keV energy were used for the final thinning of the TEM lamella for electron transparency. Phonon scattering spectra of the GeOI substrate and the GeNWs were obtained by micro-Raman spectroscopy in backscattering geometry in the range of 100 to 600 ${\mathrm{cm}}^{-1}$ using a green (532 nm) Nd:YAG laser with a liquid nitrogen-cooled charge-coupled device camera. The electrical characterization of the highly p-type doped GeOI substrate was carried out by means of a commercial *Lakeshore* Hall measurement system with van der Pauw configuration [31]. A parameter analyzer (*Agilent, 4155C*) and a superconducting magnet with fields up to ±2T were used to characterize the electrical properties of the GeNWs with Hall bar configuration.

### 2.2. Modelling

**Figure 2:**
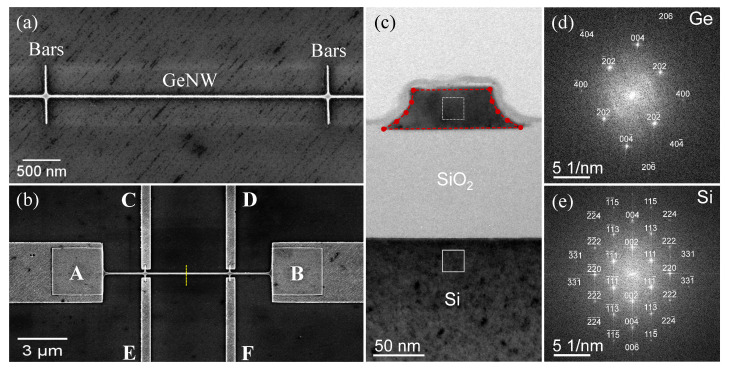
(**a**) Top-view SEM image of a 33 nm wide GeNW with narrow Ge bars; (**b**) top-view SEM image of the fabricated device with the six-contact Hall bar configuration after metal deposition. The contacts are labeled with A–F. (**c**) Cross-sectional BF-TEM image obtained from the NW location indicated in panel (**b**) by a yellow dashed line, the red dashed curve shows the cross-section considered for the simulation. (**d**,**e**) Fast Fourier transforms of HR-TEM images (not shown) from the Ge and Si region marked with a white dotted and solid square in panel (**c**), respectively. While the BF-TEM image and the Si HR-TEM image were obtained in Si $\left[1\;\bar{1}\;0\right]$ zone axis geometry (the corresponding diffractogram is indexed in panel (**e**)), the Ge HR-TEM image was recorded in Ge $\left[0\;\bar{1}\;0\right]$ zone axis geometry (the corresponding diffractogram is indexed in panel (**d**)).

The resistivity of NWs with simple shapes can be calculated analytically in case of thin films [32], circular NWs [33], and rectangular NWs [34]. Since the fabricated GeNWs showed a more complicated shape, shown in Figure 2c, we used the semi-numerical model developed by Moraga et al. [34], which can be applied to arbitrary cross-sectional shapes. This model calculates the NW resistivity from a number of classical trajectories. The average over the classical trajectories gives the characteristic function ${\langle e^{-d_{i}\;/\;\lambda_{bulk}}\rangle }_{i}$, where $\lambda_{bulk}$ is the mean free path of the bulk material and $d_{i}$ is the flight distance of the *i*-th particle, which was calculated as follows. Starting at a given position (*x*,*y*) inside the NW and a given direction of the charge carrier (ϕ,θ), the next intersection point with the surface was calculated. The reflectivity parameter *p* determined the probability of specular reflection. In case of specular reflection, the normal component of the direction was reversed and the next intersection point was calculated. With probability of 1-*p*, a diffusive scattering event occurred and the trajectory ended. A sufficiently large ensemble of trajectories was considered to obtain converged results. *p* = 0 corresponds to maximal surface roughness. The inverse of the resistivity (i.e., the conductivity) in units of the bulk resistivity followed by integrating numerically over the NW cross-section and over all possible trajectory directions as [34,35]:

(1) $${\left(\frac{\rho_{NW}}{\rho_{bulk}}\right)}^{-1}=1-\frac{3}{4\pi A}\int_{A}\;dA\;\int_{0}^{2\pi }\;d\phi \;\int_{0}^{\pi }\;d\theta \;\cos^{2}\theta \sin\theta {\langle e^{-d_{i}\;/\;\lambda_{bulk}}\rangle }_{i},$$

where $\lambda_{bulk}$ is the bulk resistivity, $\rho_{NW}$ is the NW resistivity, *A* is the cross-section of the NW, ϕ and θ are the azimuthal and polar angles, respectively. The polar axis was along the NW axis.

## 3. Results and Discussion

### 3.1. Structural Characterization

A top-view scanning electron microscopy (SEM) image of the GeNW with an average width of 33 nm with narrow Ge bars is shown in Figure 2a. Furthermore, a top-view SEM image of the fabricated device after metal deposition is shown in Figure 2b. Devices with different GeNWs widths from about 30 nm to 3 μm were fabricated. In an ideal six-contact Hall bar configuration, the contact bar pairs were located symmetrically with respect to the middle of the NWs long axis and aligned opposite to each other. The first geometrical consideration with the Hall bar configuration was the tendency of the source current contacts AB to short out the Hall voltage. If the aspect ratio of the NW length (*L*) to its width (*W*) was ≥3, then this error was less than 1% [25]. Moreover, the finite size of the contacts affected both the current density and the electrical potential in their vicinity, which could be reduced by placing metal contacts at the end of the narrow bars to avoid touching the NW sidewalls [25]. In this configuration, a current was sourced through the contacts AB, while voltage drops along the NW could be measured over the contacts CD, EF, CF, or DE for four-probe measurements to estimate the NW resistivity. For Hall effect measurements, the Hall voltage was measured via contacts CE or DF, while an external magnetic field was applied perpendicular to the substrate surface to determine the carrier concentration and mobility in the GeNWs.

To investigate the microstructure of the GeNWs, cross-sectional TEM analysis was performed. Figure 2c shows a BF-TEM image obtained from the NW location indicated with a yellow dashed line in Figure 2b. The GeNW cross-section was found to deviate from a rectangular shape due to concave sidewalls. A similar sidewall shape was observed for other fabricated GeNWs. To record the BF-TEM image in Figure 2c, the TEM specimen was oriented in Si $\left[1\;\bar{1}\;0\right]$ zone axis geometry relative to the electron beam. The fast Fourier transform obtained from an HR-TEM image of the Si region is shown in Figure 2e. Since the Ge on the SiO${}_{2}$ insulator was slightly misoriented compared to the Si substrate, the TEM specimen was tilted by several degrees to bring the GeNW in $\left[0\;\bar{1}\;0\right]$ zone axis geometry. The corresponding fast Fourier transform obtained from the HR-TEM image of the Ge region is shown in Figure 2d. It can be concluded that the GeNW is of single-crystalline structure.

**Figure 3:**
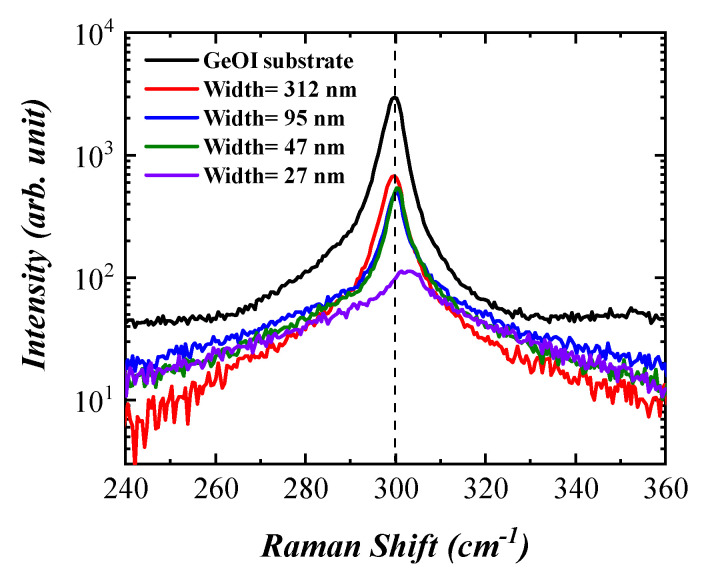
Raman spectra of GeOI substrate and GeNWs with the incident power of 3.2 mW.

Micro-Raman scattering was carried out to determine the crystallinity of the GeOI substrate and GeNWs. The Raman spectrum of the GeOI substrate, shown in Figure 3, demonstrated the transverse/longitudinal optical (TO/LO) phonon mode of Ge located at 299.7 ${\mathrm{cm}}^{-1}$. The TO/LO phonon mode in the relaxed Ge was located at 300.5 ${\mathrm{cm}}^{-1}$ [36]. The shift of the optical phonon mode toward lower wavenumbers, by about [36]. The shift of the optical phonon mode toward lower wavenumbers, by about Δω = −0.8 cm${}^{-1}$, with respect to the relaxed Ge, was related to in-plane tensile strain created in the Ge layer during the Smart-Cut process. The induced strain could be calculated using the following expression [36]:

(2) $$\varepsilon =\frac{\Delta \omega }{c}\times 100\%,$$

where Δω is the shift of the optical phonon mode position and *c* is the Raman shift coefficient, which is approximately $-300\;{\mathrm{cm}}^{-1}$ for Ge [36]. According to the peak position of the TO/LO phonon mode, the Ge layer of the GeOI substrate exhibited a tensile strain of about 0.27%.

Moreover, Raman spectra of the GeNWs with different widths are displayed in Figure 3. To avoid a heating effect, all measurements were performed with the incident power of 3.2 mW. With a decreasing NW width, the full width at half maximum increased. The peak broadening could be related to the increase in the surface-to-volume ratio and surface scattering of incident laser light. An observed blue shift of the peak from the smallest NW (27 nm) could not have been caused by a strain change or phonon confinement. Since we used a top-down approach to fabricate GeNWs and did not apply any further treatment such as implantation and annealing, the defect formation and strain engineering could be excluded. Furthermore, the phonon confinement effect of the optical phonon modes in Ge could be neglected since it appeared for GeNWs thinner than 10 nm, and phonon confinement shifted the TO phonon mode towards lower wavenumbers [37]. Here, the Raman spectrum obtained from the smallest GeNW with an average width of 27 nm located at about 302 ${\mathrm{cm}}^{-1}$ was shifted toward higher wavenumbers since it was a superposition of two phonon modes. One was related to the Si substrate, which was the second-order transverse acoustic (2TA) phonon mode [38] located at 303 ${\mathrm{cm}}^{-1}$ and the second one, much weaker, located at about 300 ${\mathrm{cm}}^{-1}$ was the TO phonon mode of 27 nm width GeNW.

### 3.2. Two- and Four-Probe Measurements

**Figure 4:**
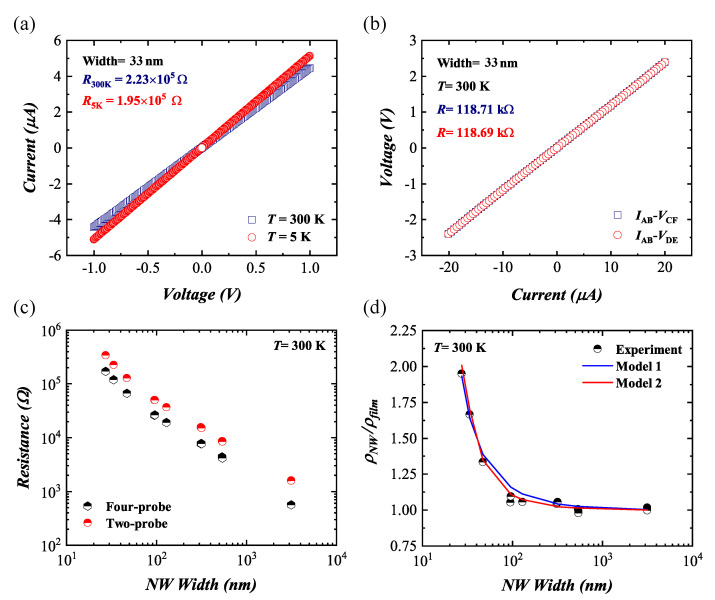
(**a**) Current–voltage curve via two-probe measurement; (**b**) voltage–current curve via four-probe measurement of GeNW with an average width of 33 nm. (**c**) Two- and four-probe resistance and (**d**) normalized GeNW resistivity as a function of NW width.

Two-probe measurements were performed to check the Ni contacts on the highly p-type doped GeNWs. Ni has already been shown to create Ohmic contacts with low specific contact resistivity [39]. The current–voltage characteristic of contacts AB is displayed in Figure 4a. As can be seen, the Ni contacts on the GeNW exhibited a linear behavior at 300 K and 5 K. All devices with different NW widths showed linear characteristics as well. In theory, low-resistance Ohmic contacts on semiconductors require metals with a low work function for n-type semiconductors and a high work function for p-type semiconductors. However, in a real device, interface states pin the Fermi level and make the barrier height independent of the metal work function [40,41]. Covalent semiconductors, such as Ge, have a large density of surface states because of unsaturated bonds at the surface. The dangling bonds and the sudden termination of the Ge crystal lattice at the interface pin the Fermi level of the semiconductor close to the valence band. Fermi level pinning at a metal/n-type Ge interface leads to the formation of a Schottky barrier independent of the metal work function. In contrast, for a metal/p-type Ge interface, Ni contacts demonstrate Ohmic behavior because of Fermi level pinning close to the valence band [40,41].

The resistance of the GeNWs was determined using four-probe measurements as shown in Figure 4b. The resistance difference between measurements on two different contact pairs was about 0.02%, proving all voltage contacts worked properly. The contact resistance was determined by a difference of two- and four-probe resistance (Figure 4c). The normalized NW resistivity with respect to the film resistivity was plotted as a function of NW width at 300 K, shown in Figure 4d. The film resistivity was defined as the resistivity of the largest fabricated wire with a cross-section of 3000nm×38nm, which was expected to behave similar to a 2D film. The resistivity of the GeNWs is listed in Table 1. As one can see, the normalized resistivity increased with a decreasing NW width. According to the theoretical models of size-dependent electrical conductivity, the NW resistivity was mainly caused by a combination of three main carrier scattering mechanisms: background or bulk scattering, external surface scattering, and grain boundary scattering [42,43]. According to the HR-TEM analysis, the GeNWs had a single-crystalline structure. The surface-to-volume ratio of the GeNWs increased with a decreasing NWs width, leading to an enhancement of the surface scattering. Therefore, external surface scattering was the dominant mechanism for the resistivity enhancement in GeNWs with less than 100 nm width. The scattering region near the surface might have been caused by a native oxide, which gave rise to a high density of interface states. To minimize the NW resistivity, one could deposit a high-quality oxide layer such as Al${}_{2}$O${}_{3}$ and anneal the GeNW in a hydrogen-containing ambient ($H_{2}/N_{2}$ forming gas), which would reduce the interface states and terminate the dangling bonds with hydrogen [44,45].

### 3.3. Simulated Normalized Resistivity

To quantify the surface and sidewall scattering, the normalized resistivity of GeNWs was fitted, first, with a $1/W_{eff}$ behavior, as shown in Figure 4d (Model 1). The effective NW width, $W_{eff}=W-2d_{sc}$, took into account a part of the volume near the sidewalls which did not contribute to the conductivity caused by the enhanced scattering close to the sidewalls. The thickness $d_{sc}$ was determined to be about 6.5 nm.

In addition, the normalized resistivity was calculated using model two, similar to Moraga et al. [34], which was developed for metallic NWs. This model is based on the Boltzmann transport equation as described in Section 2.2. The cross-section was modeled as shown in Figure 2c. The points along the sidewalls followed a Bezier curve. The area of the resulting polygon was used to calculate the experimental resistivity from the resistance values.

**Figure 5:**
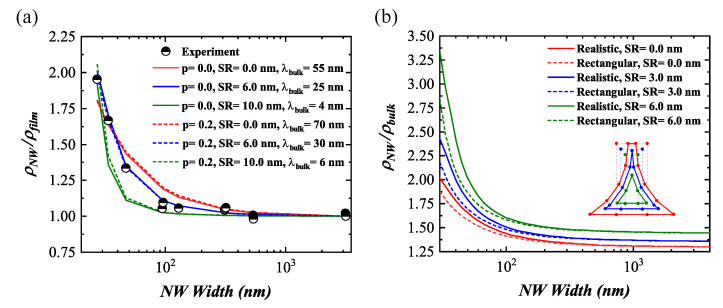
(**a**) Best fit of normalized NW resistivity for different pairs of *p* and SR. For each pair, $\lambda_{bulk}$ was determined by a least-mean-squares fit. (**b**) Normalized NW resistivity for different thicknesses of the scattering region SR and for two different shapes of the cross-section: the realistic shape and a rectangular approximation. The inset displays the corresponding cross-sectional shape for the smallest NW.

In model two, the increase in the experimentally measured resistivity could not be reproduced well, even for the best fits of reflectivity (*p*) and bulk mean free path ($\lambda_{bulk}$). Thus, we introduced an additional scattering region of thickness SR, which equally shrank the effective cross-section on all sides. Figure 5a shows the increase in the normalized resistivity for different values of parameters *p* and SR. In each case, $\lambda_{bulk}$ was obtained by a least-mean-squares fit. For *p* = 0, the best fit was observed for SR = 6 nm and $\lambda_{bulk}$ = 25 nm. A good fit could also be obtained for some other combinations of these values. However, for a too thick scattering region, e.g., SR = 10 nm, the resistivity increase occurred in the model at too small NW widths. By considering a surface with a lower roughness, i.e., *p* > 0, a similar decent fit compared to *p* = 0 could be obtained for each scattering region thickness by increasing $\lambda_{bulk}$, as shown in Figure 5a. The measurement could not be reproduced if no scattering region was considered. Therefore, we concluded that the transport in the GeNWs did not occur in the whole cross-sectional area. As shown in Figure 4d (Model 2), this approach allowed us to reproduce the experimental data for *p* = 0, $\lambda_{bulk}$ = 25 nm, and SR = 6 nm.

Figure 5b compares the simulated normalized resistivity with respect to a bulk resistivity for a realistic and rectangular approximation of the NW cross-section. The height was the same for both cross-sectional shapes. In the case of SR = 0, the width in the rectangular approximation was chosen such that the area was the same as the area of the realistic cross-section. Consequently, this was not the case for SR > 0. If the NW width was large, the rectangular approximation of the NW cross-section was found to be well suited. For small widths, however, the rectangular approximation underestimated the resistivity enhancement. These underestimations were about 7% (SR = 0) and about 14% (SR = 6 nm). The stronger resistivity increase in the realistic shape model was caused by increased scattering in the narrow edges (inset in Figure 5b). Thus, the narrow edges of the fabricated NWs were detrimental for the overall transport properties, since they were less conductive. We, further, concluded that a rectangular approximation was less suitable to model the fabricated samples.

### 3.4. Hall Effect Measurement

**Figure 6:**
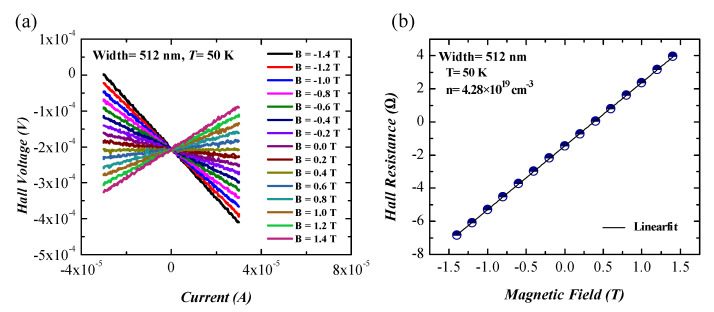
(**a**) Hall voltage–current curves at different magnetics fields; (**b**) Hall resistance as a function of magnetic field for GeNW with an average width of 512 nm.

Hall effect measurements were performed for GeNWs using the Hall bar configuration in, which a magnetic field (*B*) was oriented perpendicular to the substrate while a current was swept through the contacts AB and the Hall voltage ($V_{H}$) was, simultaneously, measured along the opposite contacts CE or DF. The Hall voltage–current curves measured at different applied magnetic fields in the range of −1.4 T to +1.4 T are shown in Figure 6a. The Hall voltage was given by [46]:

(3) $$V_{H}\;=\;\frac{1}{nq}\times \frac{IB}{t},$$

where *n* is the carrier concentration, *q* is the elementary charge, and *t* is the height of the GeNWs. The Hall resistance, obtained using the slope of the Hall voltage–current curve, as a function of an applied magnetic field, is presented in Figure 6b. Using the slope of Hall resistance vs. magnetic field and Equation (3), the carrier concentration (*n*) of the GeNWs could be estimated. The carrier Hall mobility ($\mu_{H}$) of GeNWs could be determined by obtaining the carrier concentration and resistivity (ρ), using the following Equation [46]:

(4) $$\mu_{H}\;=\;\frac{1}{n\rho q}.$$

The estimated carrier concentration and Hall mobility of GeNWs at room temperature are summarized in Table 1. One can see that the smaller GeNWs had lower carrier concentrations. This was because of the tendency of carriers to diffuse to the surface of the GeNWs and become trapped at the interface states. In general, the unpassivated GeNW is covered by a native oxide layer (GeO${}_{x}$), and the interface states of the GeNW/GeO${}_{x}$ interface act as carrier traps, which lead to a depleted region close to the NW surface. Hence, the carrier concentration of smaller GeNWs may be reduced. Moreover, the dielectric mismatch between the GeNW and the surrounding native oxide increases the ionization energy and, consequently, reduces the activation of the dopants [47,48,49].

### 3.5. Temperature-Dependent Measurement

**Figure 7:**
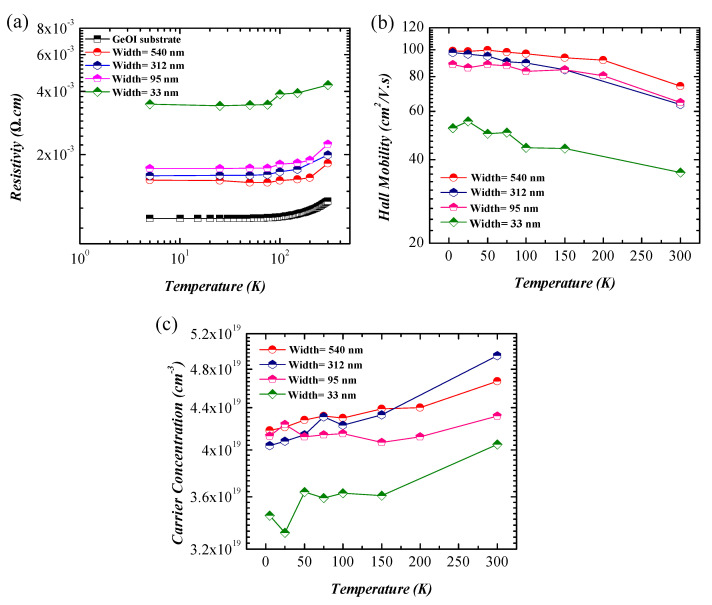
Temperature-dependent (**a**) resistivity, (**b**) Hall mobility, and (**c**) carrier concentration of GeNWs with different widths.

The temperature-dependent resistivity of the GeOI substrate and GeNWs was measured using van der Pauw geometry and Hall bar configuration, respectively. As seen in Figure 7a, the resistivity of the GeOI substrate and GeNWs decreased with a decreasing temperature, which indicated a metallic behavior. Furthermore, the temperature-dependent carrier concentration and Hall mobility values of the GeNWs are plotted in Figure 7b,c, respectively. The carrier concentration of GeNWs decreased at low temperatures because of freezing out of the intrinsic carriers. The carrier mobility was proportional to the relaxation time (τ) [46],

(5) $$\mu_{H}\;=\;\frac{q\tau }{m^{*}},$$

where $m^{*}$ is the hole effective mass. The thermal lattice vibrations decreased at low temperatures, which led to longer carrier relaxation times. Moreover, the lower carrier concentrations caused less electron–electron interactions. Therefore, the carrier mobility of the GeNWs increased at low temperatures. The GeNWs with an average width smaller than 100 nm showed lower Hall mobility values because of a higher surface-to-volume ratio, which led to higher surface scattering and, consequently, a higher resistivity. The obtained carrier mobility and its dependency on the GeNWs width was in good agreement with previous reported results [50,51,52].

## 4. Conclusions

In summary, we presented a six-contact Hall bar configuration with symmetric contact bars to characterize the electrical properties of thin GeNWs. To minimize the errors caused by intrinsic physical mechanisms, the source current was swept for each magnetic field at low temperatures.

It was shown that, by decreasing the NWs’ width, the Hall mobility decreased and the NW resistivity increased, which was related to enhanced carrier scattering at the NW surface. Two different models were used to simulate the experimental resistivity of the GeNWs. It appeared that the transport in the GeNWs did not occur in the whole cross-sectional area because of a scattering region near all NW surfaces, which reduced the effective NW cross-section.

The surface passivation of GeNWs with a high-quality oxide layer is needed to minimize the interface states, which might lead to an increase in carrier mobility. The presented symmetric Hall bar configuration for GeNWs offered a precise way regarding the Hall contacts fabrication and Hall effect measurements of thin semiconducting NWs compared to previous four-probe NW-based Hall devices.

## Figures and Tables

**Table 1:** Resistivity (ρ), carrier concentration (*n*), carrier mobility ($\mu_{H}$), and average relaxation time of carriers ($\tau_{p}$) at room temperature.

NW Width (nm)	$\rho \;(10^{-3}\;\Omega \cdot$cm)	$n\;(10^{19}$ cm${}^{-3})$	$\mu_{H}\;\left({\mathrm{cm}}^{2}V^{-1}s^{-1}\right)$	$\tau_{p}\;\left(10^{-15}\;s\right)$
3156	1.87	4.28	78.0	9.3
540	1.81	4.67	73.8	8.8
312	1.99	4.94	63.3	7.5
95	2.24	4.32	64.4	7.7
33	4.29	4.05	35.9	4.3

## Data Availability

It is not applicable.
